# Musculoskeletal pain is associated with restless legs syndrome in young adults

**DOI:** 10.1186/s12891-015-0765-1

**Published:** 2015-10-14

**Authors:** Stijn J. Hoogwout, Markus V. Paananen, Anne J. Smith, Darren J. Beales, Peter B. O’Sullivan, Leon M. Straker, Peter R. Eastwood, Nigel McArdle, David Champion

**Affiliations:** 1 Faculty of Medical Sciences, University of Groningen, PO Box 72, 9700 AB Groningen, The Netherlands; 2 Medical Research Center Oulu, Oulu University Hospital and University of Oulu, P.O. Box 8000, FI-90014 Oulu, Finland; 3 School of Physiotherapy and Exercise Science, Curtin University, GPO Box U1987, Perth, Western Australia 6845 Australia; 4 Centre for Sleep Science, School of Anatomy, Physiology & Human Biology, University of Western Australia, 35 Stirling Highway, Crawley, WA 6009 Australia; 5 Department of Anaesthesia and Pain Medicine, Sydney Children’s Hospital, High Street, Randwick, NSW 2031 Australia; 6 University of New South Wales, UNSW, Sydney, NSW 2052 Australia; 7 Offenbachlaan 14, 2253 CR Voorschoten, The Netherlands

**Keywords:** Musculoskeletal pain, Restless legs syndrome, Sleep disorders

## Abstract

**Background:**

In recent years, there is considerable evidence of a relationship between the sensorimotor disorder restless legs syndrome (RLS) and pain disorders, including migraine and fibromyalgia. An association between multi-site pain and RLS has been reported in adult women. In the current study, we explored the association between musculoskeletal (MSK) pain and RLS in a large cohort of young adults.

**Methods:**

Twenty two year olds (*n* = 1072), followed since birth of part of the Western Australian Pregnancy Cohort (Raine) Study, provided data on MSK pain (duration, severity, frequency, number of pain sites). RLS was considered present when 4 diagnostic criteria recommended by the International Restless Legs Syndrome Study Group were met (urge to move, dysaesthesia, relief by movement, worsening symptoms during the evening/night) and participants had these symptoms at least 5 times per month. Associations between MSK pain and RLS were analyzed by multivariable logistic regression with bias-corrected bootstrapped confidence intervals, with final models adjusted for sex, psychological distress and sleep quality.

**Results:**

The prevalence of RLS was 3.0 % and MSK pain was reported by 37.4 % of the participants. In multivariable logistic regression models, strong associations were found between RLS-diagnosis and long duration (three months or more) of MSK pain (odds ratio 3.6, 95 % confidence interval 1.4–9.2) and reporting three or more pain sites (4.9, 1.6–14.6).

**Conclusions:**

Different dimensions of MSK pain were associated with RLS in young adults, suggestive of shared pathophysiological mechanisms. Overlap between these conditions requires more clinical and research attention.

## Background

Restless legs syndrome (RLS) is a neurological disorder that causes an irresistible urge to move the legs, often accompanied by unpleasant sensations. The symptoms typically begin or worsen during periods of rest or inactivity, are worse during evening or night and are partially or totally relieved by movement [1]. Large epidemiologic surveys on RLS report prevalence estimates of between 4–7 % in adults [2, 3] and 2–4 % in children and adolescents [4–6]. In the absence of diagnosis and appropriate management, consequences of RLS include sleep disruption, increased daytime drowsiness, cognitive problems and reduced quality of life [2, 7]. Moreover, several studies have found high rates of comorbid psychiatric disorders in RLS [8–10] and associations between RLS and cardiovascular disease [11–13].

In recent years, there has been emerging evidence of a clinically relevant relationship between RLS and pain disorders. Associations between RLS and multiple pain problems have been identified, including migraine [14] and fibromyalgia [15, 16]. A recent Swedish population-based study among middle-aged women found a strong relationship between RLS and multi-site pain, and this association increased with both pain severity and the number of pain sites [17]. The strength of the association highlights the possibility of common causal pathways. Current theories on RLS pathophysiology emphasize brain iron deficiency leading to altered dopaminergic metabolism, combined with a strong genetic background [18]. This functional dysregulation of dopaminergic circuitry has been suggested by Stehlik et al. [17] as a mechanism in both RLS and chronic multi-site pain.

The aim of the current study was to explore the association between RLS and musculoskeletal (MSK) pain in a community-based sample of young adults, while controlling for important confounding variables as sleep quality and negative emotional symptoms. This study will be among the first investigating the association between MSK pain and RLS within this age group. Based on the previous literature, we hypothesized that the association between RLS and MSK pain will be greater in individuals with severe MSK pain and with multiple pain sites.

## Methods

### Study population

Cross-sectional data were obtained from the participants of the Western Australian Pregnancy Cohort (Raine) Study (www.rainestudy.org.au) at the age of 22. This long-term project began as a pregnancy cohort in which 2,900 women attending antenatal clinics at a tertiary level obstetric hospital in Perth, Western Australia were enrolled between 1989 and 1991. After birth, 2,868 children remained with the study to form the Raine Study cohort. An analysis at the year 17 follow-up showed that the Raine cohort had high similarity to the Western Australian population on demographic characteristics [19]. The year 22 follow-up was conducted between March 2012 and July 2014. The study sample consisted of all active members of the cohort (*n* = 2,086 or 72.7 % of the original cohort of 2,868), that is those who had previously provided consent to be contacted for follow-up and were not deceased (*n* = 40), had not withdrawn (*n* = 566), or otherwise been lost to follow-up (*n* = 176) [20]. A comparison of the active participants with census data collected in 2011 on all similarly aged young adults in Western Australia showed that the sample remains representative on a range of variables including education level, employment status, income, marital status, number of offspring, hours work and occupation. Around the time of their 22nd birthday, participants were contacted by telephone and had the details of this follow-up explained. Those wishing to participate were mailed questionnaires, participant information and a consent form. 1,072 participants (51.4 % of active participants) provided data for the variables used in this study. Ethics approval was granted from the Human Research Ethics Committees at the University of Western Australia (approval number = RA/4/1/5202) and Curtin University (HR67/2013). The study was conducted in accordance with the Declaration of Helsinki and all participants provided informed consent.

### Data collection

#### Musculoskeletal pain

Questions on MSK pain were based on the Orebro Musculoskeletal Pain Questionnaire [21]. Presence of MSK pain was assessed from 10 possible pain areas (neck, upper back, lower back, left/right shoulder, left/right arm, left/right leg and other) and four categories related to the number of pain sites were created: 1) no pain, 2) one pain site, 3) two pain sites and 4) three or more pain sites. The duration of the pain problem was defined as either of 1) short duration (less than three months) or 2) long duration (three months or more). The average pain intensity during the last week and last three months was assessed with a 11-point numerical pain rating scale with answer options ranging from ‘no pain’ to ‘pain as bad as it could be’ and categorized as 1) no pain (0–1), 2) mild pain (2–4), 3) moderate pain (5–6) or 4) severe pain (7–10). Average pain frequency during the last three months was also assessed with a 11-point numerical pain rating scale with answer options ranging from ‘never’ to ‘always’ and was classified as 1) no pain (0–1), 2) low frequency (2–4), 3) medium frequency (5–6) and 4) high frequency (7–10).

#### Restless legs syndrome

Based on the recommendations of the International Restless Legs Syndrome Study Group (IRLSSG) [1], four screening questions were used to assess the prevalence of RLS-related symptoms: 1) strong urge to move the legs when sitting or lying down, 2) accompanying dysaesthesia, 3) relief by movement and 4) worsening of symptoms during the evening/night. Individuals who answered all four questions positively and had symptoms at least 5 times per month were assigned a questionnaire-based RLS-diagnosis [22, 23].

#### Sleep quality

The subjective sleep quality component of the Pittsburgh Sleep Quality Index [24] was used as an indicator for sleep quality and four categories of sleep quality were assigned: 1) very good, 2) fairly good, 3) fairly bad and 4) very bad.

#### Anxiety/depression/stress

Data for anxiety, depression and stress were obtained using the shortened version of the Depression, Anxiety and Stress Scale (DASS-21) [25]. Although originally developed to measure psychological burden along the separate axes of depression, stress and anxiety, the DASS-21 total score can be used as a composite measure of negative emotional symptoms [25] and was used in this manner.

#### Smoking and body mass index (BMI)

Smoking status was assessed with a single question (‘Do you currently smoke cigarettes/cigars?’) and participants were classified accordingly as smokers or non-smokers. Height and weight were measured and BMI was calculated.

### Statistical analyses

Pearson’s Chi square test was applied to test differences in RLS-diagnosis prevalence across sex and MSK pain (presence of any pain, pain duration, pain severity, pain frequency and number of pain areas). Trends within distributions were tested by means of the Pearson’s Chi square test for trend.

A multivariable logistic regression analysis was performed with RLS-diagnosis as the dependent variable. Odds ratios (OR) with bias-corrected bootstrapped 95 % confidence intervals (CI) using 1000 replications were calculated for each MSK pain variable separately and the participants with no RLS-diagnosis were used as the reference group. Confounding variables were identified and incorporated into the models. The covariates used in the final regression models were sex, DASS-21 total score and sleep quality. An additional univariable analysis was performed for the separate pain regions. Since numbers per pain region were low, multivariable analysis per pain region was not performed.

All statistical analyses were performed with IBM-SPSS version 22 software (Chicago, IL, USA). A *p*-value of <0.05 was considered statistically significant.

## Results

The characteristics of the study sample, with the reference group compared to the RLS-diagnosis group, are shown in Table 1.

**Table 1:** Characteristics of the study population (*n* = 1072)^a^

	Reference group (*n* = 1040)	RLS-diagnosis group (*n* = 32)	Total (*n* = 1072)	*p*-value (for trend)
Sex				0.096
Female	560 (53.8 %)	22 (68.8 %)	582 (54.3 %)	
Male	480 (46.2 %)	10 (31.2 %)	490 (45.7 %)	
Smoking	167 (16.1 %)	6 (18.8 %)	173 (16.2 %)	0.693
Mean Body Mass Index (kg/m^2^) (SD)	25.2 (5.5)	25.1 (4.4)	25.2 (5.5)	0.936
Sleep Quality				0.105 **(0.023)**
Very good	160 (15.7 %)	3 (9.7 %)	163 (15.5 %)	
Fairly good	619 (60.6 %)	15 (48.4 %)	634 (60.3 %)	
Fairly bad	217 (21.3 %)	11 (35.5 %)	228 (21.7 %)	
Very bad	25 (2.4 %)	2 (6.5 %)	27 (2.6 %)	
Mean DASS-21 scores (SD)				
Depression Subscale	6.8 (8.2)	12.0 (11.5)	7.0 (8.4)	**0.017**
Anxiety Subscale	4.7 (5.7)	10.0 (8.0)	4.9 (5.8)	**0.001**
Stress Subscale	9.0 (8.1)	15.5 (11.0)	9.3 (8.3)	**0.002**
Total score	20.5 (19.5)	37.0 (28.3)	21.0 (20.0)	**0.003**
Any pain	380 (36.5 %)	21 (65.6 %)	401 (37.4 %)	**0.001 (0.001)**
Duration of pain				**0.001 (<0.001)**
No pain	660 (63.5 %)	11 (34.4 %)	671 (62.7 %)	
Short duration	143 (13.8 %)	5 (15.6 %)	148 (13.8 %)	
Long duration	236 (22.7 %)	16 (50.0 %)	252 (23.5 %)	
Pain intensity last week				**0.007 (0.001)**
No pain	670 (64.7 %)	11 (35.5 %)	681 (63.8 %)	
Mild pain	192 (18.5 %)	9 (29.0 %)	201 (18.8 %)	
Moderate pain	118 (11.4 %)	8 (25.8 %)	126 (11.8 %)	
Severe pain	56 (5.4 %)	3 (9.7 %)	59 (5.5 %)	
Pain intensity last 3 months				**0.016 (0.001)**
No pain	682 (65.8 %)	13 (41.9 %)	695 (65.1 %)	
Mild pain	169 (16.3 %)	6 (19.4 %)	175 (16.4 %)	
Moderate pain	105 (10.1 %)	6 (19.4 %)	111 (10.4 %)	
Severe pain	80 (7.7 %)	6 (19.4 %)	86 (8.1 %)	
Pain frequency last 3 months				**0.006 (0.001)**
No pain	683 (65.9 %)	12 (38.7 %)	695 (65.1 %)	
Low	164 (15.8 %)	6 (19.4 %)	170 (15.9 %)	
Medium	84 (8.1 %)	6 (19.4 %)	90 (8.4 %)	
High	105 (10.1 %)	7 (22.6 %)	112 (10.5 %)	
Number of pain sites				**0.005 (0.001)**
No pain	660 (63.5 %)	11 (34.4 %)	671 (62.6 %)	
One	165 (15.9 %)	8 (25.0 %)	173 (16.1 %)	
Two	106 (10.2 %)	5 (15.6 %)	111 (10.4 %)	
Three or more	109 (10.5 %)	8 (25.0 %)	117 (10.9 %)	

^a^Mean values with standard deviation provided for continuous data; numbers with percentages are provided for categorical data. Pearson’s Chi square test and Chi square test for trend were used for comparisons of categorical data, independent samples t-tests for continuous data. Significant *p*-values are highlighted in bold. *DASS-21* Depression-, Anxiety and Stress Scale, *RLS* restless legs syndrome, *SD* standard deviation

### RLS prevalence

All four RLS-diagnosis criteria were met in 32 participants, giving a prevalence of 3.0 % (Table 2). The prevalence in females was almost twice as high as in males (3.8 vs 2.0 %), but this difference did not reach statistical significance (*p* = 0.096). The 95 % confidence interval estimate for difference in prevalence between males and females was 0 to 3.7 %. Subjects with RLS reported significantly poorer sleep quality compared with the reference group (p for trend = 0.023; Table 1). Moreover, the RLS-diagnosis group had significantly higher scores on all MSK pain variables and higher DASS-21 scores. No significant differences in smoking status and BMI were found.

**Table 2:** Prevalence of restless legs related symptoms within the total cohort (*n* = 1072)

RLS symptoms assessed with 4 criteria	Female	Male	Total
Urge to move the legs (at least 5 times/month)	158 (27.1 %)	142 (29.0 %)	300 (28.0 %)
Accompanied by dysaesthesia	57 (9.8 %)	31 (6.3 %)	88 (8.2 %)
Relieved by movement	95 (16.3 %)	70 (14.3 %)	165 (15.4 %)
Worsening at evening or night	104 (17.9 %)	51 (10.4 %)	155 (14.5 %)
RLS diagnosis (All 4 RLS criteria fulfilled)	22 (3.8 %)	10 (2.0 %)	32 (3.0 %)
RLS: Restless legs syndrome			

### Prevalence of musculoskeletal pain

Current MSK pain was reported by 37.4 % of the study sample (Table 1). A short duration of pain (less than three months) was reported by 13.8 %, whereas pain of long duration (three months or more) was found in 23.5 %. The mean pain intensity score during the last three months for those reporting pain was 4.6 (SD 2.1), and severe pain was reported by 8.1 % of the subjects. The most commonly reported pain areas were the lower back (20.1 %) and neck (15.0 %) area (Table 3).

**Table 3:** Prevalences of musculoskeletal pain per region and odds ratios for RLS diagnosis^a^

	Reference group (*n* = 1040)	RLS-group (*n* = 32)	Total (*n* = 1072)	Odds ratios	*p*-value
Lower back	204 (19.6 %)	11 (34.4 %)	215 (20.1 %)	2.1 (1.0–4.3)	**0.031**
Neck	151 (14.5 %)	10 (31.3 %)	161 (15.0 %)	2.7 (1.1–6.3)	**0.024**
Upper back	93 (8.9 %)	6 (18.8 %)	99 (9.2 %)	2.4 (0.9–5.8)	0.066
Leg	82 (7.9 %)	8 (25.0 %)	90 (8.4 %)	3.9 (1.5–10.0)	**0.005**
Shoulder/arm	17 (1.6 %)	3 (9.4 %)	20 (1.9 %)	6.2 (1.9–20.8)	**0.003**
Other	62 (6.0 %)	1 (3.1 %)	63 (5.9 %)	0.5 (0.2–1.3)	0.169

^a^Odds ratios are given with bias-corrected bootstrapped 95 % confidence intervals. *P*-values of significant associations are highlighted in bold. *RLS* restless legs syndrome

### Associations between MSK pain and RLS

Univariable analysis per MSK pain region showed that all pain regions, except for the upper back region and the ‘other’ region, were significantly associated with RLS (Table 3). The strongest associations were with pain reported in the shoulder/arm region (odds ratio 6.2, 95 % confidence interval 1.9–20.8) and pain reported in the leg (3.9, 1.5–10.0). In multivariable logistic regression analysis, BMI and smoking status were tested for confounding, but were not added to the regression models, as they were not significantly associated with both MS pain and RLS. Interaction between sex and MSK pain variables was tested but did not prove to be significant. Final regression models were adjusted for sex, DASS-21 scores and sleep quality and significant associations between several MSK pain characteristics and RLS were found (Table 4). RLS was associated with reporting long duration of pain (3.6, 1.4–9.2), and medium (3.9, 1.2–12.7) pain frequency in the last three months. In addition, RLS tended to associate with mild (3.0, 1.0–8.2) and moderate pain intensity (3.6, 1.1–12.3) during the last week. Lastly, RLS was associated with both reporting one pain site (3.1, 1.1–8.5) and three or more pain sites (4.9, 1.6–14.6).

**Table 4:** Odds ratios for RLS diagnosis with musculoskeletal pain variables as the input variable^a^

	Unadjusted	Adjusted for sex	Adjusted for sex and DASS-21	Adjusted for sex, DASS-21 and sleep quality
Any body pain	*n* = 1072	*n* = 1072	*n* = 1047	*n* = 1030
No	REF	REF	REF	REF
Yes	3.3 (1.5–7.2)**	3.1 (1.5–6.7)**	2.8 (1.2–6.6)*	3.0 (1.3–7.1)*
Pain duration	*n* = 1071	*n* = 1071	*n* = 1046	*n* = 1029
No pain	REF	REF	REF	REF
Short duration	2.1 (0.6–7.4)	2.0 (0.6–7.1)	1.9 (0.6–6.4)	2.2 (0.6–7.7)
Long duration	4.1 (1.8–9.3)**	3.8 (1.7–8.6)**	3.3 (1.4–7.5)**	3.6 (1.4–9.2)**
Pain intensity last week	*n* = 1067	*n* = 1067	*n* = 1044	*n* = 1027
No pain	REF	REF	REF	REF
Mild pain	2.9 (1.1–7.4)*	2.7 (1.1–7.0)*	2.7 (1.0–7.0)*	3.0 (1.0–8.2)*
Moderate pain	4.1 (1.6–10.7)**	4.0 (1.6–10.2)**	3.3 (1.1–10.1)*	3.6 (1.1–12.3)*
Severe pain	3.3 (0.9–12.1)	2.8 (0.8–10.4)	2.7 (0.8–9.4)	2.9 (0.8–10.0)
Pain intensity last 3 months	*n* = 1067	*n* = 1067	*n* = 1044	*n* = 1027
No pain	REF	REF	REF	REF
Mild pain	1.9 (0.6–5.6)	1.8 (0.6–5.4)	1.6 (0.6–5.0)	1.8 (0.6–5.4)
Moderate pain	3.0 (0.9–9.9)	2.9 (0.9–9.3)	2.5 (0.9–7.4)	2.8 (0.8–10.0)
Severe pain	3.9 (1.3–11.5)*	3.5 (1.2–10.3)*	3.0 (1.0–8.6)*	3.2 (0.9–10.6)
Pain frequency last 3 months	*n* = 1067	*n* = 1067	*n* = 1044	*n* = 1027
No pain	REF	REF	REF	REF
Low	2.1 (0.7–5.8)	2.0 (0.7–5.7)	1.9 (0.7–5.4)	2.1 (0.7–6.5)
Moderate	4.1 (1.3–13.1)*	3.8 (1.2–12.2)*	3.5 (1.2–10.4)*	3.9 (1.2–12.7)*
High	3.8 (1.4–10.4)**	3.4 (1.3–9.4)*	2.9 (1.0–8.8)	3.2 (1.0–10.4)
Number of pain sites	*n* = 1072	*n* = 1072	*n* = 1047	*n* = 1030
No pain	REF	REF	REF	REF
One site	2.9 (1.2–7.3)*	2.8 (1.1–7.1)*	2.8 (0.9–8.4)	3.1 (1.1–8.5)*
Two sites	3.1 (0.9–10.0)	2.9 (0.9–9.1)	2.4 (0.7–7.8)	2.6 (0.8–8.9)
Three or more	6.1 (2.0–18.5)**	5.5 (1.8–16.9)**	4.5 (1.4–15.3)*	4.9 (1.6–14.6)**

^a^Odds ratios are given with 95 % Confidence Intervals. The DASS-21 total score showed strong correlation with the three subscales and was used as a continuous covariate in the multivariable logistic regression analysis. * = *p* <0.05; ** = *p* <0.01. *RLS* restless legs syndrome, *DASS-21* Depression-, Anxiety and Stress Scale

## Discussion

This study demonstrated a significant association between MSK pain and RLS in young adults from a community cohort. Specifically, strong associations were found for long duration of pain and a greater number of pain sites. These associations remained present after adjusting for sex, psychological distress and self-reported sleep quality.

### MSK pain and RLS

The current study is, to our knowledge, the first on the association between MSK pain and RLS in young adults. Most previous studies on this topic have focused on female populations and have often used chronic pain or rehabilitation clinics to recruit their participants [15, 16, 26]. In addition, these studies have focused on fibromyalgia, a more specific subset of chronic widespread pain. Our study includes both sexes, is among the first to sample from a general population and involves a more general measure of MSK pain. The questionnaires used covered multiple aspects of MSK pain and therefore provide a thorough assessment of different pain aspects. Lastly, adjustment was made for important potential confounding factors.

The results of this study are consistent with the findings by Stehlik et al. [17] in their study of Swedish middle-aged women, where long duration of pain and a high number of pain sites was strongly associated with RLS. A study in Australian twin families by Champion [27] also showed significant associations between two or more regional pain sites and RLS. These findings are, together with the results from our study, an extension to the earlier reported associations between RLS and fibromyalgia [15, 16].

Psychological distress and sleep quality are important factors in the relationship between pain and RLS. In particular, pain in multiple body areas is often related to depressive symptoms and other mental health problems including anxiety disorders [28–30]. Depression is also linked to altered pain thresholds and a predictor for future pain [31]. Several studies have established strong associations between RLS and psychiatric comorbidity including major depressive disorder and anxiety disorders [8, 32, 33]. Sleep disruption is a prominent complaint in RLS and is also considered a risk factor for both panic disorder and depression [10, 34]. High prevalence of sleep disruption has been reported in chronic MSK pain [35] and experimental studies in animals [36] and humans [37] have shown that sleep disruption can induce musculoskeletal sensitization and lower pain thresholds. In summary, the relationship between pain and sleep can be considered bidirectional [38], with an important mediating role for emotional states. In our study, the associations between RLS and MSK pain remained present even after adjusting for psychological distress and sleep quality. This underlines the probability of other shared mechanistic pathways in these conditions, which will be discussed based on the current literature.

The influence of heritable factors in RLS is supported by family studies [39, 40] and genome-wide association studies (GWAS) have identified at least six genetic loci in association with RLS [41]. Observational studies suggest, at least in part, a genetic basis in chronic pain syndromes including chronic widespread pain and fibromyalgia and pain reporting behavior at multiple MSK pain sites as well [42–44]. In GWAS a number of genes, involved in adrenergic and serotonergic pathways, have been consistently linked to MSK pain [45, 46]. Even though specific shared genes between MSK pain and RLS have not been identified thus far, it is evident that with genetic susceptibility being such an important component in the occurrence of both RLS and MSK pain, shared genes (pleiotropy) might be an important factor in the co-existence of these conditions.

The considerable role of dopamine dynamics in RLS is illustrated by the efficacy of dopamine agonists in RLS treatment [47] and supported by neuropathological studies [48]. Combined with studies establishing the link between brain iron deficiency and RLS [49, 50], it is hypothesized that RLS might be seen primarily as an iron insufficiency disorder, with resulting dopaminergic abnormalities [18]. Altered dopaminergic responses have been found in brain imaging studies of fibromyalgia patients as well [51] and reductions of dopamine metabolism have been found in patients with depression [52]. Trials with dopamine agonist pramipexole, frequently used in RLS treatment, have had promising results in both fibromyalgia patients [53] and patients with depression [54]. It is likely that alterations in dopamine metabolism are an important factor to explain the associations between RLS, MSK pain and depression.

In our study, RLS was not only strongly associated with leg pain, significant associations were also found for shoulder and arm pain, neck pain and lower back pain. An explanation for this finding might be found in the concept of central sensitization of nociception. Central sensitization is characterized by an increase in excitability and synaptic efficacy of neurons in central nociceptive pathways, manifests as pain hypersensitivity, and has been implied as a causal or maintaining factor for pain in a wide range of pathologies including osteoarthritis, temporomandibular disorders, fibromyalgia, tension-type headache, irritable bowel syndrome and low back pain [55, 56]. Recently, there has been evidence reported for central sensitization in RLS. In an interesting study by Stiasny-Kolster et al. [57] a three- to fourfold increase in sensitivity for pinprick stimuli in untreated RLS-patients was found, along with a reduced tactile sensitivity and paradoxical heat sensation. Treatment with L-Dopa reversed or reduced these symptoms, prompting the authors to conclude that sensory symptoms in RLS are possibly explained by impaired dopaminergic control on spinal nociceptive neurons and central dis-inhibition. The presence of central sensitization or dis-inhibition in RLS sufferers might account for the high comorbidity of multiple site MSK pain.

In summary, we think genetic factors might account for a large part of dysregulation in dopaminergic metabolism, involved in the onset of RLS and MSK pain, but also depression. In turn, the sleep disruption caused by RLS results in lowering of pain thresholds and an increase of depressive symptoms, both related to an increased risk of future pain. The intimate relationship between these conditions requires the clinician to have special attention for comorbid occurrence.

### Limitations

Although we acknowledge the modest response rates (51.4 %) in our study, one needs to bear in mind that the data used for this study were part of an extensive cohort study, involving questionnaires, physical assessments and for most participants also an overnight sleep study [20]. In our experience, young adults were a particularly challenging group to recruit for this study. Further, as the sample remains representative of the broader Western Australian population of similarly aged young adults, we are confident the associations found are likely to be true for the population. The RLS-categorization was based on self-reported measures, which might increase prevalence estimates compared to a clinician-based diagnosis. We used the IRLSSG [1] screening questions in conjunction with frequency of experiencing symptoms at least 5 times per month as conservative criteria for diagnosis of RLS. Alternate questionnaires for RLS diagnosis, such as the Cambridge-Hopkins questionnaire [58], have been suggested as an alternative to the 4 screening questions based on their ability to better differentiate disorders that could mimic RLS [59]. However in the large epidemiological Raine Study where participants provided extensive questionnaire datasets, the 4 screening questions were deemed sufficient to make the RLS diagnosis while minimising participant burden. Also the likelihood of common RLS mimics being present in the younger cohort, including conditions as neuropathy, radiculopathy and arthritic pains [59], was deemed low. Despite these potential limitations we still found strong, consistent associations. Even though we did not find a statistically significant difference, the RLS prevalence was almost twice as high in females as in males in our samples, consistent with previous reports [2, 3, 5]. The 95 % confidence interval for the difference in RLS-prevalence between males and females from our sample was 0 to 3.7 %, in keeping with the known female predominance. Also, the overall prevalence of RLS found in this study is congruent with other large epidemiological surveys [2–6] supporting the representativeness of the Raine cohort and the conservative criteria used for RLS diagnosis. Most known confounders, based on previous literature, were controlled for, but we cannot be certain that all potential confounders were measured. Because of the relatively small group that satisfied the RLS criteria, confidence intervals in our risk estimations were wide. Larger studies are needed if a more accurate estimation of RLS-prevalence risk in MSK pain is sought. Lastly, while we found strong associations between RLS and MSK pain, the cross-sectional nature of our study limits the possibility to make statements on causality. Future studies, particularly prospective studies, are needed to effectively address this causality question.

### Clinical implications

We have found strong independent associations between MSK pain and RLS in young adults. This finding indicates that there may be an opportunity for early intervention and prompt clinicians to screen for RLS in young adults with MSK pain, and vice versa. Each condition should be regarded in the light of the other. Consideration of the comorbidity of MSK pain and RLS, as well as addressing psychological symptoms such as depression, may be particularly important in determining management needs. In subjects showing an overlap between RLS, multiple pains and depressive symptoms, dopamine agonists such as pramipexole might prove especially effective, as already has been shown for these conditions separately [47, 53, 54]. While pharmalogical treatment is the frontline approach for RLS [47], in RLS sufferers with comorbid MSK pain non-pharmalogical strategies such as exercise programs [60] might be a useful adjunct. Intervention studies are needed to further investigate treatment options in this particular category of patients.

## Conclusions

In conclusion, our study demonstrates associations of RLS with long duration of MSK pain and a high number of pain sites in a community-based setting in young adults. The underlying shared mechanisms might be related to sleep disruption, psychological distress, genetic factors, dopaminergic dysfunction or central sensitization. Additional research is needed to clarify the mechanisms behind these associations and to improve treatment options in patients suffering simultaneously from these two conditions.

## Abbreviations

BMI: Body mass index
DASS-21: Depression-, Anxiety and Stress Scale
IRLSSG: International restless legs syndrome study group
MSK: Musculoskeletal
RLS: Restless legs syndrome
SPSS: Statistical package for the social sciences
